# Polymorphism Analysis of *NOTCH2* and *CD1A* Genes and Their Association with Wool Traits in Subo Merino Sheep

**DOI:** 10.3390/biology14101336

**Published:** 2025-09-28

**Authors:** Shengchao Ma, Wenna Liu, Asma Anwar, Sen Tang, Yaqian Wang, Gulinigaer Aimaier, Cuiling Wu, Xuefeng Fu

**Affiliations:** 1 Xinjiang Key Laboratory of Special Species Conservation and Regulatory Biology, International Center for the Collaborative Management of Cross-Border Pest in Central Asia, College of Life Science, Xinjiang Normal University, Urumqi 830017, China; shengchaomasicau@163.com (S.M.); lwn2362@163.com (W.L.); 13579812147@163.com (G.A.); 2 Xinjiang Key Laboratory of Animal Biotechnology, Key Laboratory of Genetic Breeding and Reproduction of Herbivorous Livestock of Ministry of Agriculture and Rural Affairs, Xinjiang Uygur Autonomous Region Academy of Animal Science, Urumqi 830011, China; asma247462@163.com (A.A.); tangsensen610@163.com (S.T.); wangyaqlan@163.com (Y.W.)

**Keywords:** Subo Merino sheep, *NOTCH2* gene, *CD1A* gene, wool traits, polymorphism

## Abstract

To identify molecular markers associated with wool traits in fine-wool sheep, we examined genetic polymorphisms in the *NOTCH2* and *CD1A* genes in 944 Subo Merino sheep in this study. Subsequently, we performed association analyses between mutation sites in the *NOTCH2* and *CD1A* genes and wool traits using SAS 9.4 software, followed by linkage disequilibrium (LD) analysis of different mutation sites using Haploview 4.2 software. Additionally, bioinformatics tools were employed to predict the potential impacts of missense mutations on protein secondary and tertiary structures. Finally, quantitative PCR (qPCR) was used to assess the expression levels of the *NOTCH2* and *CD1A* genes. Genetic analysis revealed six polymorphic sites in *NOTCH2* and *CD1A*, all of which were missense mutations. Two SNPs in *NOTCH2* (SNP1 and SNP2) showed significant associations with the coefficient of variation of fibre diameter, and SNP1 was also associated with greasy fleece weight. Four SNPs in *CD1A* (SNP3–SNP6) were significantly associated with fibre diameter standard deviation, and SNP3, SNP4, and SNP5 were additionally associated with crimp number. LD analysis revealed that SNP3, SNP4, and SNP5 were closely linked. Bioinformatics analysis indicated that the mutations caused alterations in the secondary and tertiary structures of the *NOTCH2* and *CD1A* proteins. qPCR results showed that the *CD1A* gene was highly expressed in the fine wool fibre group compared with the ultra-fine wool fibre group. In conclusion, this study revealed a genetic association between *NOTCH2* and *CD1A* and wool traits. The results are expected to provide a theoretical foundation for breeding wool traits in Subo Merino sheep, thereby enhancing the economic value of fine wool.

## Simple Summary

To identify the genetic factors influencing wool quality in Subo Merino sheep, this study focused on the *NOTCH2* and *CD1A* genes. Through genetic analysis, we detected six single nucleotide polymorphisms (SNPs) within these genes and examined their associations with key wool traits. Our results indicated that specific SNPs in *NOTCH2* are closely linked to the coefficient of variation of fibre diameter and greasy fleece weight, while SNPs in *CD1A* are significantly associated with the standard deviation of fibre diameter and crimp number. We also found that these genetic variations may alter the secondary and tertiary structures of the proteins encoded by *NOTCH2* and *CD1A*, potentially affecting their biological functions. Furthermore, qPCR results demonstrated that *CD1A* is higher expressed in fine wool, reinforcing its potential role in regulating wool quality. Overall, this research identifies promising candidate SNPs that could serve as molecular markers, providing practical guidance for breeding Subo Merino sheep with improved wool traits and enhancing our understanding of the genetic mechanisms underlying wool quality.

## 1. Introduction

Fine-wool sheep are predominantly found in the pastoral and semi-pastoral regions of central and western China, as well as in ethnic border areas. They play a vital role in Xinjiang’s animal husbandry and have become a key industry for improving the livelihoods of local farmers and herders [1]. However, in recent years, the fine-wool sheep industry in Xinjiang has faced significant challenges due to the increasing dominance of meat sheep production. The widening price gap between meat and wool has reduced profitability in fine-wool breeding, leading to declining wool output, low breeding efficiency, and inferior wool quality. Although domestic wool is influenced by many factors, as a high-grade raw material in the textile industry, the long-term viability of wool is an inevitable trend [2]. Currently, in the wool market, the price of ultrafine wool is two to three times that of ordinary wool. Therefore, high-quality fine wool is the focus of market attention and a key target for scientific research breakthroughs [3]. However, traditional breeding methods can no longer meet the demands of wool production. To accelerate the development of the wool industry, molecular breeding is particularly important in the breeding and selection of fine-wool sheep [4].

Single nucleotide polymorphisms (SNPs) are considered the most promising molecular markers due to their abundance, wide distribution, and rich informational content. With the continual reduction in the costs of chip and sequencing technologies, SNP marker technology has been widely adopted in animal breeding research. The polymorphisms of numerous genes and their associations with wool traits have gradually been elucidated. Studies have identified significant associations between specific SNP variants in the *PTPN3*, *KRT83*, *KRT39*, *CCSER1*, *RPS6KC1*, *KCNRG*, *KCNK9*, and *CLYBL* genes and wool or cashmere traits. These genes may serve as valuable candidates for improving wool and cashmere quality, providing important references for breeding programmes and research into genetic mechanisms [5,6,7,8].

In preliminary studies, our research group identified several candidate genes (*FZD3*, *ARPP21*, *LMNB1*, *RASA1*, *PAK1*, *IFNAR2*, *FAT3*, *CD1A*, and *NOTCH2*) through genome-wide association analysis [9]. Notably, members of the Notch family play a crucial role in hair follicle morphogenesis, regulating the proliferation and differentiation of follicular cells across multiple developmental stages. Specifically, *NOTCH1*, *NOTCH2*, and *NOTCH3* exhibit distinct spatial expression patterns within different hair follicle cell layers [10,11,12]. He et al. [13] demonstrated that *NOTCH1* expression peaks at embryonic day 105 (E105) during ovine skin development, suggesting its pivotal role in folliculogenesis. Complementary findings by Vauclair et al. [14] confirmed *NOTCH1*’s essential functions in late-stage embryonic follicle development and postnatal hair cycle regulation. Similarly, *NOTCH2* has been implicated in cutaneous differentiation and follicular development [15]. Regarding CD1A, this transmembrane protein, a member of the CD1 family, plays dual roles in immune regulation and antigen presentation. As a dominant skin antigen-presenting molecule, CD1A-targeted neutralising antibodies show therapeutic potential for dermatological and systemic disorders [16]. Recently, it has been found that changes in CD1A-dependent T cell responses are associated with the pathogenesis of various inflammatory skin diseases [17]. Therefore, we conclude that the distribution of *CD1A* in skin immune cells is closely related to the pathological state of the skin, which may affect the immune environment surrounding hair follicles. In summary, the *NOTCH2* and *CD1A* genes play important roles in hair growth and can be used as candidate genes affecting fine wool traits for SNP mining.

Subo Merino sheep, a novel superfine-wool breed developed in 2014, exhibit remarkable stress resistance, high reproductive survival rates, superior wool characteristics (17–19 μm fibre diameter), and high wool yield. This breed has filled China’s gap in fine-wool sheep production (80 s wool grade), enriched the nation’s fine-wool germplasm resources, and enhanced both domestic wool quality and international market competitiveness [18,19,20]. Therefore, in this study, the SNPs of *NOTCH2* and *CD1A* genes were identified in Subo Merino sheep, and the genetic effects of these SNPs on wool traits were analysed in depth, aiming to provide new markers for the molecular breeding of wool traits in fine-wool sheep.

## 2. Materials and Methods

### 2.1. Phenotype Measurement and Sample Collection

In this study, total of 944 one-year-old Subo Merino ewes were obtained from two regions in Xinjiang: Yili Gongnaisi Sheep Farm (*n* = 473) and Aksu Baicheng Sheep Farm (*n* = 471). Sample collection involved the following four steps:

(1) Blood samples were collected from the jugular vein of these 944 Subo Merino sheep using heparin anticoagulant tubes. After heparin is mixed with blood, the blood samples were immediately placed in an icebox, further transported to the laboratory, and finally stored at −20 °C refrigerator for DNA extraction;
(2) While taking blood samples, the greasy fleece weight (GFW), live weight before shearing (LWBS), and live weight after shearing (LWAS) of these sheep were measured and recorded. Additionally, the staple length (SL), fineness count (FC), crimp, hair length (HL), and crimp number (CN) of these sheep were also measured and recorded [21];
(3) Wool samples were collected from a site 10 cm posterior to the left scapular edge (midline region). Wool samples were further washed using the conventional washing process and allowed to dry naturally. Measurements of the mean fibre diameter (MFD), coefficient of variation of fibre diameter (CVFD), and fibre diameter standard deviation (FDSD) were taken in a laboratory maintained at a constant temperature (20 ± 2 °C) and humidity (65 ± 4%) using a fibre diameter optical analyser (OFDA2000, Ningbo Jiangnan Instrument Factory, Ningbo, China) [22]. The parameters measured included. Excel 2019 was used to compile the data on wool traits. SPSS 27.0 software [23] was employed to perform descriptive statistical analyses on the relevant wool trait data;
(4) Based on the results of MFD measurements, 10 sheep with the smallest MFD were designated as the ultra-fine wool fibre group (UFW, 16.21 ± 0.46 μm), and 10 with the largest MFD as the fine wool fibre group (FW, 20.68 ± 0.93 μm). For these two groups, additional wool samples were collected from the left forelimb and 5 cm posterior to the scapula and were further used to measure the MFD. 20 skin tissue samples (approximately 2 cm × 2 cm) from these two groups were collected using a skin sampler, immediately frozen in liquid nitrogen, and were further stored at −80 °C refrigerator for RNA extraction.

### 2.2. DNA Extraction and SNP Typing

Total of 944 blood genomic DNA was extracted using the Blood/Cell/Tissue Genomic DNA Extraction Kit (DP304) from Tiangen Biochemical Technology (Beijing) Co., Ltd. (Beijing, China). The quality of the DNA was assessed by 1.0% agarose gel electrophoresis, and its concentration was measured using an NanoDrop™ 2000 devices (Thermo Fisher Scientific, Waltham, MA, USA). Samples with a concentration greater than 20 ng/μL and an OD260/OD280 ratio between 1.7 and 1.9 were deemed suitable for the experiment. Subsequently, SNP typing was performed using Fluidigm’s Biomark™ HD system (Biomark™ HD, San Francisco, CA, USA).

### 2.3. Genetic Diversity Analysis

Excel 2019 was utilised to organise the data and to calculate genotype frequencies, allele frequencies, and genetic diversity parameters, including homozygosity (Ho), heterozygosity (He), effective number of alleles (Ne), polymorphism information content (PIC), and Hardy–Weinberg equilibrium for each genotype within the population [24,25]. Additionally, when the Hardy–Weinberg equilibrium test yields a *p* > 0.05, it indicates that the population is in Hardy–Weinberg equilibrium.

The calculation formulas for Ho, He, Ne, PIC and $X^{2}$ are as follows:

(1) $$Ho=\sum_{i=1}^{m}P_{i}^{2}$$

(2) $$He=1-\sum_{i=1}^{m}P_{i}^{2}$$

(3) $$Ne=\frac{1}{\sum_{i=1}^{m}P_{i}^{2}}$$

(4) $$PIC=1-\sum_{i=1}^{m}P_{i}^{2}-\left(\sum_{i=1}^{m-1}\sum_{j=i+1}^{m}2P_{i}^{2}P_{j}^{2}\right)$$

(5) $$X^{2}=\sum_{i}^{n}\frac{{\left(\left|O_{i}-E_{i}\right|-1/2\right)}^{2}}{E_{i}}$$

where *m* is the number of alleles, $P_{i}$ and $P_{j}$ are the frequencies of the *i*-th and *j*-th alleles in the population, $E_{i}$ is the expected frequency, and $O_{i}$ is the observed frequency.

Subsequently, linkage disequilibrium (LD) analysis of the mutation sites was conducted using Haploview 4.2 software [26]. Finally, haplotype analysis was carried out using the geneHapR package [27] in the R language 4.4.2.

### 2.4. Correlation Analysis

According to the results of wool fibre diameter measurements, the correlation between different genotypes and wool traits was analysed using the GLM procedure in SAS 9.4 [28]. The least-squares variance analysis method was employed, with genotype and field effects treated as fixed effects. During the GLM analysis, *t*-tests were conducted between levels. Concurrently, Tukey’s method was employed for multiple comparison correction. The results are presented as least-squares means ± standard error. *p* < 0.05 indicates a significant difference, and *p* < 0.01 indicates a highly significant difference.

The linear model is as follows:

(6) $$Y_{ick}=\mu +G_{i}+F_{c}+e_{ick}$$

In the form, $Y_{ick}$: sheep individual phenotypic value; μ: group mean $G_{i}$: genotypic SNP effect; $F_{c}$: field effect; $e_{ick}$: random error.

### 2.5. Biological Function Prediction

Non-synonymous SNPs result in changes to the protein sequence before and after mutation. Using information on different genotypes at non-synonymous mutation sites, the wild-type and mutant gene sequences at these sites in the *NOTCH2* and *CD1A* genes were retrieved from the Ensembl nucleic acid database. SOPMA [29] was employed to predict the secondary structure of the protein before and after mutation, while SWISS-MODEL [30] was used to predict the tertiary structure, with all parameters set to default.

### 2.6. RNA Extraction

Total RNA was extracted using the TRIzol method. The OD260/280 and OD260/230 ratios of the total RNA were measured with a NanoDrop™ 2000 devices (Thermo Fisher Scientific, MA, USA), and the integrity of the RNA (RIN) was assessed using an Agilent 2100 Bioanalyser (Agilent Technologies, Santa Clara, CA, USA). The OD260/280 ratio ranged from 1.8 to 2.0, the OD260/230 ratio was greater than 2.0, and the RIN value ranged from 7.0 to 8.5, indicating suitability for subsequent experiments. Next, reverse transcription was performed using the Takara reverse transcription kit (DRR036A) from Bao Bioengineering (Dalian) Co., Ltd. (Dalian, China).

### 2.7. Primer Design and qPCR

According to the NCBI database, the sheep *NOTCH2* gene sequence (accession number: XM_060401816.1) and the *CD1A* gene sequence (accession number: XM_042256151.1) were obtained. Primer pairs were designed using Primer Premier 5.0 and validated via NCBI Primer-BLAST (https://blast.ncbi.nlm.nih.gov, accessed on 30 May 2025) to select suitable candidates for the assay. The primers were then commercially synthesised by Bioengineering (Shanghai) Co., Ltd. (Shanghai, China). Simultaneously, the *GAPDH* gene was used as the internal reference gene [31]. The list of primers is shown in Table 1. Subsequently, the reaction conditions for real-time PCR were established by referring to the system and parameters recommended in the Talent qPCR PreMix (SYBR Green) kit manual (FP209) from Tiangen Biochemical Technology (Beijing) Co., Ltd. (Beijing, China). qPCR detection was performed using the CFX96™ Real-Time System instrument (Bio-Rad, Hercules, CA, USA). The amplification procedure was as follows: pre-denaturation at 95 °C for 3 min, followed by denaturation at 95 °C for 5 s, annealing at 60 °C for 10 s, and extension at 72 °C for 15 s, with fluorescence signal collection. The last three steps were repeated 40 times, followed by a dissociation curve stage. The qPCR results were calculated using the 2^−ΔΔCt^ algorithm [32].

## 3. Results

### 3.1. Descriptive Statistics of Wool Traits

Descriptive statistics for wool traits in Subo Merino sheep were calculated, and the results are presented in Table 2. The MFD was 17.71 μm, ranging from 12.80 to 24.00 μm, with FDSD of 1.84 μm and CVFD of 10.39%. The average values and ranges of each trait correspond with objective data, and the small standard deviation indicates that the variables are relatively concentrated around the mean.

### 3.2. Analysis of Mutation Sites in the NOTCH2 and CD1A Genes

Six mutation sites were identified in the *NOTCH2* and *CD1A* genes of Subo Merino sheep, all of which were missense mutations. SNP1 is located in exon 30 of the *NOTCH2* gene, and SNP2 is located in exon 24 of the *NOTCH2* gene, with A → C and G → A mutations, respectively. SNP3 is located in the second exon of the *CD1A* gene, with a G → A mutation. SNP4 and SNP5 are located in the third exon of the *CD1A* gene, with A → C and G → A mutations, respectively. SNP6 is located in the fourth exon of the *CD1A* gene, with an A → T mutation. Detailed information on the specific mutation sites is provided in Table 3.

### 3.3. Analysis of Genotype Frequencies and Allele Frequencies of NOTCH2 and CD1A Genes

As shown in Table 4, for SNP1 of the *NOTCH2* gene, the GG genotype and G allele were predominant, whereas for SNP2, the CC genotype and C allele were predominant. For the *CD1A* gene, SNP3 and SNP5 shared the same predominant genotype (GG) and allele (G); SNP4 was characterised by the AA genotype and A allele; and SNP6 was characterised by the TT genotype and T allele. The chi-square test indicated that SNP1, SNP2, SNP3, SNP4, and SNP5 were in Hardy–Weinberg equilibrium (*p* > 0.05), whereas SNP6 deviated from Hardy–Weinberg equilibrium (*p* < 0.05).

### 3.4. Population Genetic Analysis of NOTCH2 and CD1A Genes

The genetic diversity of SNPs in Subo Merino sheep was analysed, and the results are presented in Table 5. As shown, the Ho for the SNPs in the *NOTCH2* and *CD1A* genes were 0.739, 0.783, 0.559, 0.556, 0.554, and 0.753, respectively. The Ne for these SNPs were 1.36, 1.285, 1.737, 1.741, 1.764, and 1.797, respectively. With regard to PIC, the values for SNP3, SNP4, SNP5, and SNP6 ranged between 0.25 and 0.5, indicating moderate polymorphism. In contrast, the PIC values for SNP1 and SNP2 were less than 0.25, corresponding to low polymorphism at these loci. These results suggest that the *CD1A* gene loci exhibit relatively high polymorphism.

### 3.5. Genetic Effects of NOTCH2 and CD1A Genes on Wool Traits

#### 3.5.1. Variance Analysis of Different Genotypes of *NOTCH2* and *CD1A* Genes on Wool Traits

The effects of SNPs in the *NOTCH2* and *CD1A* genes on the wool traits of Subo Merino sheep were analysed using least-squares analysis of variance. The results are presented in Table 6. SNP1 had a significant effect on MFD (*p* < 0.05), SNP2 significantly affected the CVFD (*p* < 0.05), and SNP4 significantly influenced SL (*p* < 0.05). SNP6 had a significant effect on FDSD and CVFD in this group (*p* < 0.05), while the remaining SNPs showed no significant effects (*p* > 0.05).

#### 3.5.2. Association Analysis of *NOTCH2* and *CD1A* Genes with Wool Traits

Table 7 presents the results of association analyses between SNPs in the *NOTCH2* and *CD1A* genes and wool traits. For FDSD, significant associations were observed with SNP3, SNP4, and SNP5 (*p* < 0.05), a highly significant association with SNP6 (*p* < 0.01), and no significant associations with SNP1 or SNP2 (*p* > 0.05). MFD, SL, FC, LWAS, and LWBS showed no significant associations with any SNPs (*p* > 0.05). Regarding SNP3, individuals with GA or GG genotypes exhibited significant associations with HL and crimp compared to those with the AA genotype (*p* < 0.05), while AA or GA genotypes were significantly associated with CN compared to the GG genotype (*p* < 0.05). For SNP4, AA and AC genotypes were significantly associated with crimp relative to the CC genotype (*p* < 0.05), and CN differed significantly between AC and CC genotypes (*p* < 0.05). For SNP5, AA and GA genotypes showed significant associations with CN compared to the GG genotype (*p* < 0.05). In SNP6, AT and TT genotypes were significantly associated with CVFD relative to the AA genotype (*p* < 0.05). Regarding SNP1, CVFD differed significantly among GG, TG, and TT genotypes (*p* < 0.05), and GFW in the TT genotype showed significant differences compared to the GG and TG genotypes (*p* < 0.05). In SNP2, CVFD in the CC genotype differed significantly from that in the TT and TC genotypes (*p* < 0.05), while no significant associations were detected between other wool traits and genotypes (*p* > 0.05).

### 3.6. LD and Haplotype Analysis

**Figure 1:**
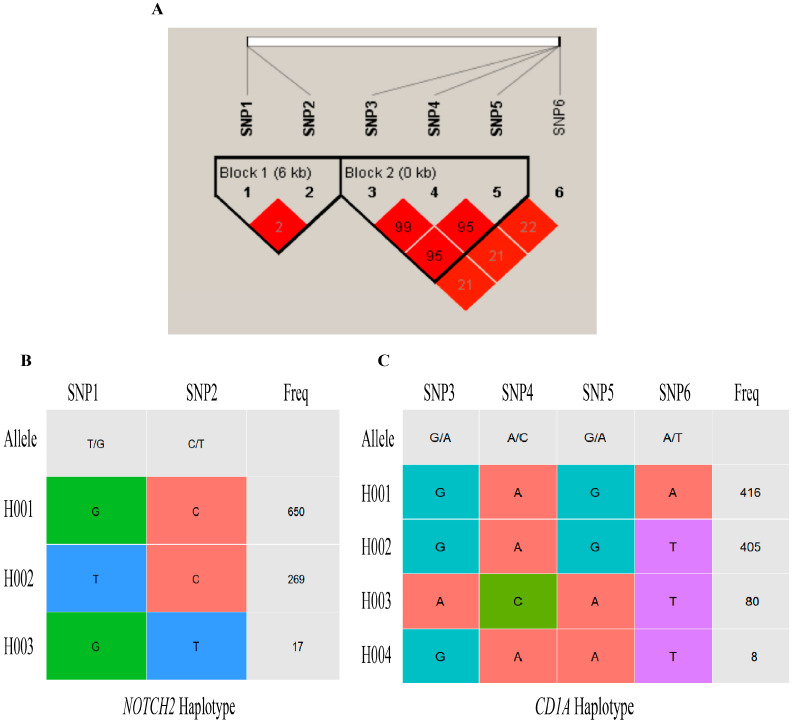
Haplotype block diagram. (**A**) *NOTCH2* and *CD1A* genes SNPs loci haplotype block distribution; (**B**) *NOTCH2* gene haplotype analysis, the alleles of *NOTCH2* SNPs (SNP1: T/G; SNP2: C/T) form distinct haplotypes (H001, H002, H003), with Freq indicating the frequency of each haplotype in the studied population; (**C**) *CD1A* gene haplotype analysis, the alleles of *CD1A* SNPs (SNP3: G/A; SNP4: A/C; SNP5: G/A; SNP6: A/T) form distinct haplotypes (H001, H002, H003, H004).

LD analysis was conducted on six SNPs of the *NOTCH2* and *CD1A* genes using Haploview 4.2 software. The linkage between SNPs is illustrated in Figure 1A. The colour of each block ranges from light to dark (white to red), indicating the degree of linkage from low to high; a darker colour signifies stronger linkage. The D′ and r^2^ values range from 0 to 1, with higher values indicating a greater degree of linkage. An r^2^ value of 1 denotes complete LD, while r^2^ > 0.6 indicates strong LD. As shown in Table 8, the D’ values for SNP1 and SNP2, SNP3 and SNP4, SNP3 and SNP5, and SNP4 and SNP5 are all 1, indicating a high correlation and close linkage between these SNP pairs.

A haplotype refers to the combination of alleles at multiple loci that are inherited together on the same chromosome. In the Subo Merino sheep population, three haplotypes of the *NOTCH2* gene were identified (Figure 1B). Among these, the H001 haplotype, primarily GC, had a frequency of 69.44%; H002, primarily TC, had a frequency of 28.74%; and H003 was mainly GT. Additionally, four haplotypes were observed in the *CD1A* gene (Figure 1C). The H001 haplotype, predominantly GAGA, had a frequency of 45.76%; H002, mainly GAGT, had a frequency of 44.55%; H003 was primarily ACAT; and H004 was mainly GAAT.

### 3.7. Analysis of Protein Structure Changes

**Figure 2:**
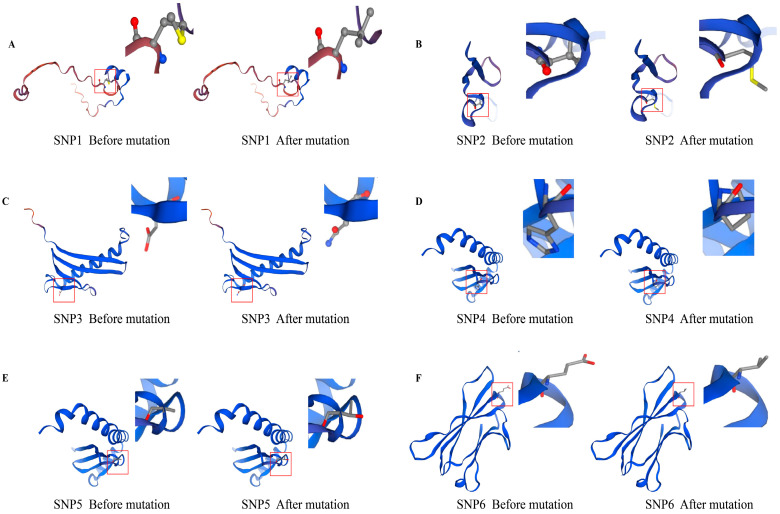
Prediction of protein tertiary structure before and after mutation of 6 sites. (**A**) Comparison of SNP1 tertiary structure before and after mutation; (**B**) Comparison of SNP2 tertiary structure before and after mutation; (**C**) Comparison of SNP3 tertiary structure before and after mutation; (**D**) Comparison of SNP4 tertiary structure before and after mutation; (**E**) Comparison of SNP5 tertiary structure before and after mutation; (**F**) Comparison of SNP6 tertiary structure before and after mutation. The red box indicates the position before and after the SNP mutation.

An analysis of the amino acid substitutions at the mutation sites and their impact on protein secondary structure revealed that the proteins encoded by the *NOTCH2* and *CD1A* genes comprise three types of secondary structures, primarily random coils, α-helices, and extended strands (Table 9). The mutation in SNP1 resulted in the amino acid at position 1805 changing from methionine to leucine, which led to a decrease in the proportion of α-helices and an increase in the proportion of extended strands. The mutation in SNP2 caused the amino acid at position 1307 to change from valine to methionine, resulting in a reduction in the proportion of random coils and an increase in extended strands. The mutation in SNP4 led to the substitution of histidine by proline at position 123, causing an increase in the proportion of α-helices and a decrease in random coils. The mutation in SNP6 resulted in the amino acid at position 270 changing from glutamic acid to valine, which increased the proportion of random coils and decreased the proportion of extended strands. The mutation in SNP3 replaced aspartic acid at position 78 with asparagine, while the mutation in SNP5 substituted alanine at position 127 with threonine. However, these two mutations did not alter the proportions of the protein’s secondary structures. Homology modelling of the protein tertiary structures before and after the missense mutations, performed using SWISS-MODEL, showed that the predicted tertiary structures were consistent with the secondary structure predictions, with random coils predominating in the protein structure (Figure 2).

### 3.8. qPCR Results of NOTCH2 and CD1A Genes

**Figure 3:**
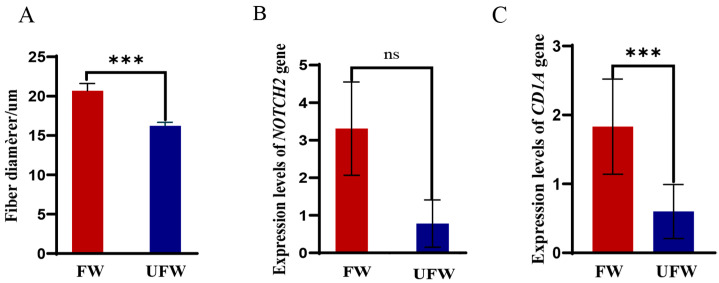
qPCR results of *NOTCH2* and *CD1A* genes. (**A**) Fibre diameter comparison between FW and UFW groups; (**B**) *NOTCH2* gene expression levels in FW vs. UFW groups; (**C**) *CD1A* gene expression levels in FW and UFW groups. ***: *p* < 0.01, ns: *p* > 0.05.

Based on wool fibre diameter measurements, the ten individuals with the lowest MFD were classified as the FW (mean: 20.68 ± 0.93 μm), while the ten with the highest MFD were designated as the UFW (mean: 16.21 ± 0.46 μm). As shown in Figure 3, the expression levels associated with different MFD in Subo Merino sheep exhibited highly significant differences between the UFW and FW (*p* < 0.01, Figure 3A). Furthermore, the expression levels of the *NOTCH2* and *CD1A* genes were analysed in both groups. The results demonstrated that both genes were expressed at higher levels in the FW compared to the UFW. However, while *NOTCH2* showed no statistically significant difference (*p* > 0.05, Figure 3B), *CD1A* exhibited a higher significant differential expression (*p* < 0.01, Figure 3C). In conclusion, the *NOTCH2* and *CD1A* genes may contribute to the observed differences in MFD between the two groups.

## 4. Discussion

The growth and development of wool are regulated by the expression of related genes at the molecular level, with variations in wool traits resulting from mutations in key genes [33]. Studies by Ma et al. [34,35], Wang et al. [36], Zhao et al. [37], and Yue et al. [38] have demonstrated, through genome-wide association studies and gene polymorphism validation, that SNPs in genes such as *ALX4*, *keratin-associated protein 2-1*, *KIF16B*, *SLIT3*, and *ZNF280B* are associated with traits including SL, crimp, MFD, and wool production. In summary, analysing the genetic mechanisms underlying wool quality at the molecular level is of great significance for the breeding of fine-wool sheep.

Studies have shown that Notch signalling pathway is one of the top ten gene families implicated in hair follicle development and villus growth [39]. Bai et al. [40] analysed the correlation between *NOTCH2* gene polymorphisms and villus and growth traits in Shaanbei white cashmere goats. Their results demonstrated that two mutation sites, rs665021370 and rs653705114, in the *NOTCH2* gene had significant effects on villus and growth traits. Dilinare [41] investigated the genetic effects of the *KRT85*, *NOTCH2*, and *ADAM9* genes on wool traits in Chinese Merino sheep (Xinjiang type). The findings revealed that the g.96438799 C>T locus of the *NOTCH2* gene had an extremely significant effect on SL (*p* < 0.01), and the g.96432471 T>G locus had an extremely significant effect on CN (*p* < 0.01). The results of this study strongly support the significant role of the *NOTCH2* gene in regulating wool traits. Specifically, *NOTCH2* gene SNPs 1 and 2 were significantly associated with CVFD, while SNP1 was significantly associated with GFW. However, no significant association was found between the *NOTCH2* gene and CN or SL, which is inconsistent with the findings of Dilinare [41]. We therefore speculate that this discrepancy may be attributable to variations in breed, sample size, and rearing conditions. To explore this further, qPCR analysis was conducted. Although *NOTCH2* gene expression tended to be higher in the FW group, no significant difference was observed between the FW and UFW groups. This suggests that *NOTCH2* gene may not play a major role in regulating MFD at the transcriptional level in this context. Instead, its potential influence on CVFD or GFW is likely mediated through post-transcriptional mechanisms or gene interactions. Since the *CD1A* gene was identified through GWAS screening and functional prediction as influencing the fineness of fine-wool sheep in our earlier work [9], further exploration of the *CD1A* gene is warranted. The qPCR results of this study also support the association of the *CD1A* gene with wool MFD. Mitchell et al. [42] identified that *CD1A* T cells may be involved in the pathology of various diseases, including cancer and autoimmune disorders. The results of this study showed that SNP3, SNP4, and SNP5 of the *CD1A* gene were significantly correlated with FDSD and CN, while SNP6 was significantly correlated with FDSD. SNP3 was significantly associated with HL (*p* < 0.05). Additionally, SNP3 and SNP4 were significantly associated with crimp (*p* < 0.05), and SNP6 was significantly associated with CVFD. These findings further support the important role of the *CD1A* gene in regulating wool traits.

PIC and heterozygosity are indicators used to assess genetic polymorphism within a population [43]. In the Subo Merino sheep population, the PIC values for the two mutation sites in the *NOTCH2* gene ranged from 0.197 to 0.230, indicating low polymorphism. In contrast, the four mutation sites in the *CD1A* gene exhibited PIC values between 0.334 and 0.345, reflecting moderate polymorphism and suggesting a moderate level of genetic variation with relatively abundant polymorphism. Notably, SNP6 was found to deviate from Hardy–Weinberg equilibrium, which may indicate that factors such as natural selection, genetic drift, non-random mating, or mutation are influencing the population’s gene frequencies. This warrants further investigation into the causal factors and their impact on the population’s adaptability and viability. LD analysis revealed strong LD between SNP1 and SNP2, SNP3 and SNP4, SNP3 and SNP5, and SNP4 and SNP5, indicating non-random allelic associations and close linkage due to their physical proximity on the same chromosome, making them less likely to separate during genetic transmission. Additionally, this study found that SNP6 was in LD with other loci. This phenomenon may result from the unique genetic effect of this locus or the presence of other linked loci acting together on wool traits. Therefore, it is necessary to evaluate these SNPs separately, considering their different linkage relationships, to determine their breeding value and specific applications in breeding practices.

The spatial structure of proteins is closely related to their function and provides valuable biological information [44]. Studies have shown that SNPs located in gene exons can directly cause changes in the encoded amino acids, thereby affecting protein structure, function, and expression levels [45]. Existing research has confirmed that missense mutations can influence phenotypes by altering protein structure [46]. For example, missense mutations in the *TLR2* gene can affect immune function [47]; missense mutations in the *MYO7B*, *KIF13B*, and *LOC101121854* genes are associated with sheep body weight [48]; and missense mutations in the *KRTAP36-2* and *FST* genes regulate wool yield and wool fibre diameter, respectively [49,50]. Therefore, it is necessary to investigate the relationship between changes in the secondary and tertiary structures of *NOTCH2* and *CD1A* gene proteins and wool-related traits. In this study, mutations in the *NOTCH2* gene at SNP1 and SNP2 resulted in changes such as a decrease in the proportion of α-helices, an increase in the proportion of extended strands, and a decrease in the proportion of random coil. As *NOTCH2* is involved in signalling pathways related to hair follicle development, changes in its protein structure may disrupt the accuracy and efficiency of signal transmission, ultimately affecting hair follicle morphogenesis, cyclic growth, and wool traits such as hair thickness and density. The secondary structure of the *CD1A* gene remained unchanged before and after mutations at the SNP3 and SNP5 sites. However, following mutations at the SNP4 and SNP6 sites, the secondary structure underwent changes including an increase in the proportion of α-helices, a decrease in the proportion of random coil, and a decrease in the proportion of extended strands. We speculate that these changes may affect the recognition and binding of the CD1A protein with other proteins, which is crucial for maintaining the stability of the hair follicle microenvironment.

## 5. Conclusions

In this study, six mutation sites were identified in the *NOTCH2* and *CD1A* genes of Subo Merino sheep. SNP1 and SNP2 of the *NOTCH2* gene were significantly correlated with CVFD and GFW, while SNP3, SNP4, SNP5, and SNP6 of the *CD1A* gene were significantly correlated with FDSD and CN. The results indicate that the *NOTCH2* and *CD1A* genes can influence their functions by altering the secondary and tertiary structures of the proteins. Furthermore, qPCR analysis demonstrated that the *CD1A* gene is higher expressed in the FW. In summary, the findings of this study provide a foundation for further investigation into the genetic mechanisms underlying the wool traits of Subo Merino sheep. The identified mutation sites are expected to serve as potential molecular markers affecting the wool performance of this breed and offer a reference for molecular breeding aimed at improving wool quality.

## Figures and Tables

**Table 1:** qPCR primer sequences.

Primer	Primer Sequences (5′-3′)	Product Size (bp)	Annealing Temperature (°C)
*NOTCH2*	F:GCTTCACTGGTTCCTTCTGC	119	60
	R:ATAGCCCAATGGACAGATGC		
*CD1A*	F:TGACGTCTTGCCTAATGCTG	124	60
	R:GATGATGTCCTGGCCTCCTA		
*GAPDH*	F:GGTGATGCTGGTGCTGAGTA	118	59.86
	R:CAGCAGAAGGTGCAGAGATG		

**Table 2:** Descriptive statistics of wool traits in Subo Merino sheep.

Traits	Number	Mean	Standard Deviation	Minimum	Maximum	Coefficient of Variation
MFD	944	17.71	1.84	12.80	24.00	10.39
FDSD	944	4.14	0.57	2.80	6.40	13.77
CVFD	944	23.38	2.24	17.00	30.80	9.58
SL	944	88.26	11.77	50.00	130.00	13.34
FC	937	66.82	2.52	60.00	80.00	3.77
crimp	936	1.84	0.62	1.00	3.00	33.70
HL	937	9.89	0.95	6.00	14.00	9.61
LWBS	936	33.18	5.03	22.00	50.00	15.16
LWAS	668	34.13	4.96	22.00	50.00	14.53
GFW	765	3.33	0.54	2.00	5.60	16.22
CN	932	13.89	3.00	8.00	21.00	21.60

**Table 3:** Information of SNPs of *NOTCH2* and *CD1A* genes.

Genes	SNPs	Area	Chromosome: Location	Nucleotide Variation	Amino Acid Variation
*NOTCH2*	SNP1	Exon30	1: 96432471	c. 5413A/C	p. Met1805Leu
	SNP2	Exon24	1: 96438799	c. 3919G/A	p. Val1307Met
*CD1A*	SNP3	Exon2	1: 107486485	c. 232G/A	p. Asp78Asn
	SNP4	Exon3	1: 107487136	c. 368A/C	p. His123Pro
	SNP5	Exon3	1: 107487147	c. 379G/A	p. Ala127Thr
	SNP6	Exon4	1: 107487782	c. 809A/T	p. Glu270Val

**Table 4:** Genotype frequency and allele frequency of *NOTCH2* and *CD1A* genes.

Genes	SNPs	Genotype Frequency	Allele Frequency	χ^2^	*p*
*NOTCH2*	SNP1	TT (0.03), TG (0.26), GG (0.71)	T (0.16), G (0.84)	0.702	*p* > 0.05
	SNP2	CC (0.76), CT (0.22), TT (0.02)	C (0.87), T (0.13)	0.661	*p* > 0.05
*CD1A*	SNP3	GG (0.47), GA (0.44), AA (0.09)	G (0.70), A (0.30)	0.258	*p* > 0.05
	SNP4	AA (0.47), AC (0.44), CC (0.09)	A (0.69), C (0.31)	0.216	*p* > 0.05
	SNP5	GG (0.46), GA (0.45), AA (0.09)	G (0.68), A (0.32)	0.386	*p* > 0.05
	SNP6	AA (0.21), AT (0.25), TT (0.54)	A (0.33), T (0.67)	7.86 × 10^−40^	*p* < 0.05

**Table 5:** Population genetic analysis of *NOTCH2* and *CD1A* genes.

Genes	SNPs	Ho	He	Ne	PIC
*NOTCH2*	SNP1	0.739	0.261	1.360	0.230
	SNP2	0.783	0.217	1.285	0.197
*CD1A*	SNP3	0.559	0.441	1.737	0.334
	SNP4	0.556	0.444	1.741	0.335
	SNP5	0.554	0.446	1.764	0.339
	SNP6	0.753	0.247	1.797	0.345

**Table 6:** Variance analysis of different genotypes of *NOTCH2* and *CD1A* genes on wool traits.

Genes	SNPs	MFD /μm	FDSD /μm	CVFD /%	SL /cm	FC /Count	HL /cm	Crimp	LWAS /kg	LWBS /kg	GFW /kg	CN
*NOTCH2*	SNP1	4.40 *	2.41	2.24	1.06	1.53	0.63	0.21	0.43	0.63	2.07	0.63
	SNP2	0.19	1.66	2.78 *	0.63	0.30	1.69	0.99	1.59	1.26	2.42	1.28
*CD1A*	SNP3	2.07	2.19	1.18	0.02	1.26	1.83	2.10	0.57	0.50	0.38	1.42
	SNP4	0.94	2.43	2.39	2.74 *	0.96	2.60	2.12	0.31	1.40	0.16	1.53
	SNP5	2.06	2.41	1.56	0.31	1.54	0.54	1.17	0.62	0.25	1.04	1.93
	SNP6	0.70	3.04 *	2.63 *	0.37	0.50	0.21	1.20	0.65	0.51	0.38	0.10

*: *p* < 0.05.

**Table 7:** Association of SNPs in *NOTCH2* and *CD1A* genes with wool traits.

Genes	SNPs	Genotype	MFD /μm	FDSD /μm	CVFD /%	SL /cm	FC /Count	HL /cm	Crimp	LWAS /kg	LWBS /kg	GFW /kg	CN
*NOTCH2*	SNP1	GG	17.688 ± 0.057	4.191 ± 0.023	23.437 ± 0.087 ^a^	88.447 ± 0.458	66.835 ± 0.096	10.114 ± 0.035	1.832 ± 0.025	32.826 ± 0.202	33.063 ± 0.160	3.320 ± 0.027 ^a^	13.931 ± 0.088
		TG	17.881 ± 0.094	4.230 ± 0.037	23.500 ± 0.144 ^a^	88.151 ± 0.756	66.887 ± 0.159	10.164 ± 0.058	1.864 ± 0.040	33.201 ± 0.327	33.418 ± 0.264	3.200 ± 0.046 ^b^	13.852 ± 0.146
		TT	17.710 ± 0.292	4.007 ± 0.117	22.291 ± 0.448 ^b^	84.186 ± 2.357	66.451 ± 0.494	9.919 ± 0.179	1.840 ± 0.126	32.446 ± 0.968	32.949 ± 0.820	3.228 ± 0.143	14.051 ± 0.453
	SNP2	CC	17.771 ± 0.055	4.201 ± 0.022	23.391 ± 0.083	88.457 ± 0.439	66.796 ± 0.092	10.142 ± 0.033	1.841 ± 0.023	33.001 ± 0.194	33.188 ± 0.153	3.263 ± 0.027	13.842 ± 0.085
		TC	17.699 ± 0.103	4.208 ± 0.041	23.596 ± 0.156 ^a^	87.676 ± 0.824	66.948 ± 0.173	10.056 ± 0.063	1.858 ± 0.044	32.667 ± 0.369	33.197 ± 0.287	3.356 ± 0.049	14.055 ± 0.160
		TT	17.634 ± 0.356	4.002 ± 0.142	22.422 ± 0.542 ^b^	87.795 ± 2.858	66.504 ± 0.600	10.189 ± 0.217	1.588 ± 0.152	30.434 ± 1.579	31.979 ± 0.992	3.552 ± 0.201	14.756 ± 0.549
*CD1A*	SNP3	AA	17.885 ± 0.146	4.154 ± 0.065	23.361 ± 0.252	87.994 ± 1.325	66.942 ± 0.277	10.125 ± 0.112	1.89 ± 0.070	33.269 ± 0.536	33.087 ± 0.460	3.241 ± 0.077	14.367 ± 0.254 ^a^
		GA	17.810 ± 0.072	4.253 ± 0.029 ^a^	23.571 ± 0.110	88.278 ± 0.580	66.674 ± 0.122	10.198 ± 0.049 ^a^	1.78 ± 0.031 ^a^	32.778 ± 0.260	33.033 ± 0.202	3.283 ± 0.035	13.799 ± 0.112 ^b^
		GG	17.683 ± 0.070	4.159 ± 0.028 ^b^	23.293 ± 0.107	88.292 ± 0.561	66.936 ± 0.118	10.050 ± 0.047 ^b^	1.89 ± 0.030 ^b^	32.971 ± 0.242	33.302 ± 0.196	3.298 ± 0.034	13.920 ± 0.108
	SNP4	AA	17.673 ± 0.07	4.158 ± 0.028 a	23.300 ± 0.107	88.216 ± 0.571	66.935 ± 0.119	10.122 ± 0.429	1.885 ± 0.030 ^a^	32.968 ± 0.242	33.269 ± 0.197	3.298 ± 0.034	13.926 ± 0.109
		AC	17.809 ± 0.072	4.254 ± 0.029 ^b^	23.580 ± 0.110	88.244 ± 0.576	66.689 ± 0.121	10.145 ± 0.044	1.785 ± 0.031 ^b^	32.768 ± 0.260	33.035 ± 0.201	3.281 ± 0.035	13.797 ± 0.111 ^a^
		CC	17.887 ± 0.165	4.154 ± 0.065	23.358 ± 0.251	88.013 ± 1.319	66.941 ± 0.277	9.968 ± 0.100	1.885 ± 0.070	33.272 ± 0.536	33.094 ± 0.460	3.242 ± 0.077	14.366 ± 0.254 ^b^
	SNP5	AA	17.901 ± 0.157	4.153 ± 0.062	23.355 ± 0.240	88.836 ± 1.264	66.921 ± 0.265	10.006 ± 0.096	1.850 ± 0.067	33.206 ± 0.515	33.142 ± 0.440	3.244 ± 0.073	14.360 ± 0.242 ^a^
		GA	17.812 ± 0.072	4.251 ± 0.029 ^a^	23.563 ± 0.109	88.155 ± 0.576	66.693 ± 0.121	10.137 ± 0.044	1.798 ± 0.031	32.839 ± 0.260	33.055 ± 0.201	3.288 ± 0.035	13.784 ± 0.111 ^b^
		GG	17.655 ± 0.071	4.156 ± 0.028 ^b^	23.289 ± 0.108	88.209 ± 0.570	66.914 ± 0.120	10.132 ± 0.044	1.877 ± 0.030	32.945 ± 0.244	33.274 ± 0.199	3.296 ± 0.035	13.937 ± 0.110
	SNP6	AA	17.682 ± 0.123	4.201 ± 0.049	23.462 ± 0.187	87.582 ± 0.985	66.863 ± 0.207	10.130 ± 0.075	1.867 ± 0.053	32.817 ± 0.494	33.231 ± 0.344	3.345 ± 0.652	13.828 ± 0.190
		AT	17.864 ± 0.103	4.309 ± 0.041 ^A^	23.768 ± 0.157 ^a^	88.159 ± 0.826	66.747 ± 0.174	10.119 ± 0.063	1.774 ± 0.044	33.254 ± 0.380	33.005 ± 0.289	3.299 ± 0.051	13.858 ± 0.160
		TT	17.722 ± 0.077	4.152 ± 0.030 ^B^	23.284 ± 0.117 ^b^	88.658 ± 0.617	66.813 ± 0.130	10.129 ± 0.047	1.854 ± 0.033	32.756 ± 0.275	33.158 ± 0.216	3.258 ± 0.037	13.953 ± 0.119

Note: Different uppercase letters (A or B) indicate extremely significant difference (*p* < 0.01), and different lowercase letters (a or b) indicate significant difference (*p* < 0.05).

**Table 8:** D’ (upper triangle) and r^2^ values (lower triangle) of SNPs in *NOTCH2* and *CD1A* genes.

SNPs	SNP1	SNP2	SNP3	SNP4	SNP5	SNP6
SNP1	-	1.000	0.000	0.000	0.000	0.000
SNP2	0.027	-	0.000	0.000	0.000	0.000
SNP3	0.000	0.000	-	1.000	1.000	0.972
SNP4	0.000	0.000	0.998	-	1.000	0.972
SNP5	0.000	0.000	0.954	0.956	-	0.973
SNP6	0.000	0.000	0.211	0.212	0.224	-

**Table 9:** Prediction of protein secondary structure before and after mutation of 6 sites.

Genes	SNPs	Genotype	α-Helix (%)	β-Turn (%)	Random Coil (%)	Extension Strand (%)
*NOTCH2*	SNP1	Wild type	14.29	0.00	71.43	14.29
		Mutant type	10.71	0.00	71.43	17.86
	SNP2	Wild type	0.00	0.00	73.68	26.32
		Mutant type	0.00	0.00	71.05	28.95
*CD1A*	SNP3	Wild type	49.44	0.00	33.71	16.85
		Mutant type	49.44	0.00	33.71	16.85
	SNP4	Wild type	37.63	0.00	41.94	20.43
		Mutant type	38.71	0.00	40.86	20.43
	SNP5	Wild type	37.63	0.00	41.94	20.43
		Mutant type	37.63	0.00	41.94	20.43
	SNP6	Wild type	0.00	0.00	60.22	39.78
		Mutant type	0.00	0.00	62.37	37.63

## Data Availability

The data and material used in this research are available from the corresponding author upon request.

## Abbreviations

The following abbreviations are used in this manuscript:

UFW	Ultra-fine wool fibre group
FW	Fine wool fibre group
MFD	Mean fibre diameter
CVFD	Fibre diameter variation coefficient
FDSD	Fibre diameter standard deviation
SL	Staple length
FC	Fineness count
HL	Hair length
CN	Crimp number
GFW	Greasy fleece weight
LWBS	Live weight before shearing
LWAS	Live weight after shearing
SNPs	Single nucleotide polymorphisms
LD	Linkage disequilibrium
Ho	Homozygosity
He	Heterozygosity
Ne	Effective number of alleles
PIC	Polymorphism information content
qPCR	quantitative PCR

## References

[1] Li Y. (2018). Production status and healthy development way of fine wool in China. Anim. Breed. Feed..

[2] Liu Y., Wang J., Shi G., Wan P. (2024). Current Status of Xinjiang Fine-wool Sheep Breeding and Measures for Genetic Resource Conservation and Utilization. Xinjiang Farm Res. Sci. Technol..

[3] Li W., Liu Y., Di J., Xu X. (2020). Exploring the Breeding Status and Development Trends of Fine-Wool Sheep. Jilin Anim. Husb. Vet. Med..

[4] Marhaba A., Tian Y., Bai Y., Yang X., Tian K., Huang X. (2019). A New SNP Mutation of GPR143 Gene of Chinese Merino Sheep (Xinjiang Type) and Its Genetic Effect Analysis. Grass-Feed. Livest..

[5] Lu X., Suo L., Yan X., Li W., Su Y., Zhou B., Liu C., Yang L., Wang J., Ji D. (2024). Genome-Wide Association Analysis of Fleece Traits in Northwest Xizang White Cashmere Goat. Front. Vet. Sci..

[6] Wang C., Yuan Z., Hu R., Li F., Yue X. (2023). Association of SNPs within PTPN3 Gene with Wool Production and Growth Traits in a Dual-Purpose Sheep Population. Anim. Biotechnol..

[7] Chai W., Zhou H., Forrest R.H.J., Gong H., Hodge S., Hickford J.G.H. (2017). Polymorphism of KRT83 and Its Association with Selected Wool Traits in Merino-Cross Lambs. Small Rumin. Res..

[8] Yu X. (2025). Molecular Characterization of KRT24, KRT33B, KRT39 and KRT84 Genes and Association Analysis of Their Variations with Wool Traits in Gansu Alpine Fine—Wool Sheep. Master’s Thesis.

[9] Zhao B. (2020). Screen the Differentially Expressed Genes of Skin Tissue in Fine—Wool Sheep and Analysis of Genetic Effects. Master’s Thesis.

[10] Zhang Y., Wang L., Li Z., Chen D., Han W., Wu Z., Shang F., Hai E., Wei Y., Su R. (2019). Transcriptome Profiling Reveals Transcriptional and Alternative Splicing Regulation in the Early Embryonic Development of Hair Follicles in the Cashmere Goat. Sci. Rep..

[11] Powell B.C., Passmore E.A., Nesci A., Dunn S.M. (1998). The Notch Signalling Pathway in Hair Growth. Mech. Dev..

[12] Gordon-Thomson C., Botto S.A., Cam G.R., Moore G.P.M. (2008). Notch Pathway Gene Expression and Wool Follicle Cell Fates. Aust. J. Exp. Agric..

[13] He J., Wei C., Huang X., Zhang G., Mao J., Li X., Yang C., Zhang W., Tian K., Liu G. (2024). MiR-23b and miR-133 Cotarget TGFβ2/NOTCH1 in Sheep Dermal Fibroblasts, Affecting Hair Follicle Development. Cells.

[14] Vauclair S., Nicolas M., Barrandon Y., Radtke F. (2005). Notch1 Is Essential for Postnatal Hair Follicle Development and Homeostasis. Dev. Biol..

[15] Fu X. (2022). Selection of Regulatory Factors Related to Cashmere Fiber Diameter Trait from Transcriptome and Proteome Profiles in Tibetan Cashmere Goats. Ph.D. Thesis.

[16] Hardman C.S., Chen Y.-L., Wegrecki M., Ng S.W., Murren R., Mangat D., Silva J.-P., Munro R., Chan W.Y., O’Dowd V. (2022). CD1a Promotes Systemic Manifestations of Skin Inflammation. Nat. Commun..

[17] Ye J.H., Chen Y.-L., Ogg G. (2024). CD1a and Skin T Cells: A Pathway for Therapeutic Intervention. Clin. Exp. Dermatol..

[18] Liu N., Tian K., Shi G., He J., Liu J., Di J., Yang Y. (2015). Effect of Different Generations on Wool Traits during Upgrading Cross Stages in Subo Merino Nuleus Herd. Chin. J. Anim. Sci..

[19] He J. (2023). Integrating Transcriptome and miRNA Data to Analyze the Molecular Mechanism of Hair Follicle Development in Subo Merino Sheep. Ph.D. Thesis.

[20] Ma S., Long L., Huang X., Tian K., Tian Y., Wu C., Zhao Z. (2023). Transcriptome Analysis Reveals Genes Associated with Wool Fineness in Merinos. PeerJ.

[21] Yu L., Fu X., Li W., Wang Q., Xu X., Xie M., Zhang Y., Di J. (2024). Methods on measuring and assessing major wool traits in fine wool sheep. Wool Text. J..

[22] Li Z., Zheng W., Xing W., Lu X., Zhang M., Hu X., Fan B., Quan K., Liu J. (2024). Study on the Detection Results of Fine Wool Fiber Diameter in Different Temperature and Humidity Environments. Grass Feed. Livest..

[23] Hoyt R.E., Snider D., Thompson C., Mantravadi S. (2016). IBM Watson Analytics: Automating Visualization, Descriptive, and Predictive Statistics. JMIR Public Health Surveill..

[24] Zhang J. (2021). Genetic Effect Analysis and Breeding Application of Growth Trait Genes in Ujumqin Sheep. Master’s Thesis.

[25] Liu B. (2013). Selection, Identification and SNP Analysis of Correlation Geneswith Cashmere Growth on Cashmere Goats. Ph.D. Thesis.

[26] Barrett J.C. (2009). Haploview: Visualization and Analysis of SNP Genotype Data. Cold Spring Harb. Protoc..

[27] Zhang R., Jia G., Diao X. (2023). geneHapR: An R Package for Gene Haplotypic Statistics and Visualization. BMC Bioinform..

[28] Lee J. (1987). Analysis of Covariance by the SAS GLM Procedure. Comput. Biol. Med..

[29] Geourjon C., Deléage G. (1995). SOPMA: Significant Improvements in Protein Secondary Structure Prediction by Consensus Prediction from Multiple Alignments. Comput. Appl. Biosci..

[30] Waterhouse A., Bertoni M., Bienert S., Studer G., Tauriello G., Gumienny R., Heer F.T., de Beer T.A.P., Rempfer C., Bordoli L. (2018). SWISS-MODEL: Homology Modelling of Protein Structures and Complexes. Nucleic Acids Res..

[31] Tian Y.Z., Usman T., Tian K.C., Di J., Huang X.X., Xu X.M., Tulafu H., Wu W.W., Fu X.F., Bai Y. (2017). Comparative Study of 13 Candidate Genes Applying Multi-Reference Normalization to Detect the Expression of Different Fineness in Skin Tissues of Wool Sheep. Genet. Mol. Res..

[32] Livak K.J., Schmittgen T.D. (2001). Analysis of Relative Gene Expression Data Using Real-Time Quantitative PCR and the 2^−ΔΔCT^ Method. Methods.

[33] Xu X., HanikeziI T., Liu J., Shi G., Fu X., Yu L., Lazhate A., Di J. (2022). Genome-wide association of wool length and yield of Chinese Merino sheep (Xinjiang type). J. Northwest A F Univ. (Nat. Sci. Ed.).

[34] Ma L., Tian J., Wang J., Zhao Z., Ma Q. (2025). Association of ALX4 gene with wool traits in Tan sheep and its expression in skin tissue. J. Gansu Agric. Univ..

[35] Ma L., Zhao W., Ma Q., Wang J., Zhao Z., Zhang J., Gu Y. (2024). Genome-Wide Association Study of Birth Wool Length, Birth Weight, and Head Color in Chinese Tan Sheep Through Whole-Genome Re-Sequencing. Animals.

[36] Wang J., Zhou H., Hickford J.G.H., Luo Y., Gong H., Hu J., Liu X., Li S., Song Y., Ke N. (2020). Identification of the Ovine Keratin-Associated Protein 2-1 Gene and Its Sequence Variation in Four Chinese Sheep Breeds. Genes.

[37] Zhao H., Hu R., Li F., Yue X. (2021). Two Strongly Linked Blocks within the KIF16B Gene Significantly Influence Wool Length and Greasy Yield in Fine Wool Sheep (*Ovis aries*). Electron. J. Biotechnol..

[38] Yue L., Lu Z., Guo T., Liu J., Yuan C., Yang B. (2023). Association of SLIT3 and ZNF280B Gene Polymorphisms with Wool Fiber Diameter. Animals.

[39] Lin H.-Y., Kao C.-H., Lin K.M.-C., Kaartinen V., Yang L.-T. (2011). Notch Signaling Regulates Late-Stage Epidermal Differentiation and Maintains Postnatal Hair Cycle Homeostasis. PLoS ONE.

[40] Bai J., Liu X., Liu L., Song Y., Zhang X., Song X., Shi L., Li L., Zhang L., Zhu H. (2023). Polymorphism of Notch2 Gene and Its Association Analysis with Cashmere and Growth Traits in Shaanbei White Cashmere Goats. China Anim. Husb. Vet. Med..

[41] Dilinare A. (2017). Genetic Effect Study of KRT85, Notch2 and ADAM9 Genes on Wool Traits of Chinese Merino (Xinjiang Type) Sheep. Master’s Thesis.

[42] Mitchell J., Kannourakis G. (2021). Does CD1a Expression Influence T Cell Function in Patients with Langerhans Cell Histiocytosis?. Front. Immunol..

[43] Qi J., Pei Q., Zhang W., Xu T., Zuo M., Han B., Li X., Liu D., Wang S., Zhou B. (2024). Genome-wide Selective Signal Identification and Association Analysis of Candidate Genes for Tibetan Sheep Wool Traits. Acta Vet. Et Zootech. Sin..

[44] Zhang H., Wang H., Lu R., Lan J., Chen L., He X. (2024). Advances in the application of AlphaFold2: A protein structure prediction model. Chin. J. Biotechnol..

[45] Zhang Y. (2025). Based on WGS to Analyze the Genetic Diversity and Selection Signals of 7 Meat Goat Breeds in Southwest China. Master’s Thesis.

[46] Zhu C. (2025). Screening of miRNAs and circRNAs in Yak Muscle Tissue and Correlation Analysis of ACACB Gene. Master’s Thesis.

[47] Tang S., Yang Y., Wang J., Wen M., Zhou B., Chen Z., Yue J. (2018). Polymorphism analysis of TLR2 gene in different breeds of sheep based on sequencing technology. Chin. J. Prev. Vet..

[48] Liu Z., Qin Q., Zhang C., Xu X., Lan M., Alatansuhe, Wang Z., Liu Z. (2024). Effects of 5 SNP mutation sites on the body weight of Ujimqin sheep at different time points. Sci. Sin. (Vitae).

[49] Zhou H., Li W., Bai L., Wang J., Luo Y., Li S., Hickford J.G.H. (2023). Ovine KRTAP36-2: A New Keratin-Associated Protein Gene Related to Variation in Wool Yield. Genes.

[50] Mir T., Ibrahim M., Siraj M., Bangash S.A.K., Khan S.H., Khan M., Tayyab M., Ahmad S. (2025). Novel Mutations in Exon 2 of Follistatin (FST) Gene Associated with Wool Fiber Diameter in Sheep. Small Rumin. Res..

